# A New Fluorescence Band of Anthocyanins as a Simple Oxidation Biomarker of Food Products

**DOI:** 10.3390/molecules30122510

**Published:** 2025-06-08

**Authors:** Małgorzata Rak, Grzegorz Bartosz, Izabela Sadowska-Bartosz

**Affiliations:** 1 Laboratory of Analytical Biochemistry, Institute of Food Technology and Nutrition, Faculty of Technology and Life Sciences, University of Rzeszow, 4 Zelwerowicza Street, 35-601 Rzeszow, Poland; mrak@ur.edu.pl (M.R.); gbartosz@ur.edu.pl (G.B.); 2 Doctoral School, University of Rzeszow, 16C Rejtana Street, 35-959 Rzeszow, Poland

**Keywords:** anthocyanins, anthocyanidins, fluorescence, oxidation, lipid peroxidation, blueberry, black carrot, oil

## Abstract

The formation of a new fluorescence band of anthocyanidins and anthocyanidins, centered at about 530 nm (excitation at 460–470 nm), is proposed as a simple indicator of food oxidation. This fluorescence band appeared and increased progressively during the incubation of blueberry juice under aerobic conditions and the cooking of blueberry homogenate and black carrot. The same effect was observed upon the addition of delphinidin to rapeseed oil subjected to simulated frying. A ratiometric parameter (ratio of the fluorescence intensity at the maximum of the new band to the fluorescence intensity of native anthocyanins/anthocyanidin) is proposed as a versatile index useful for the estimation of the oxidation of food products containing anthocyanins or supplemented with anthocyanins or anthocyanidins.

## 1. Introduction

Anthocyanins are a class of flavonoid compounds that mainly provide color to the flowers and fruits of many plants to attract pollinators and dispersers and contribute to the red colors of many autumn leaves [1,2]. In plants, anthocyanins have antioxidant functions and play an important role in scavenging reactive oxygen species. Moreover, they act as sunscreens, protecting plants against light and UV radiation. They may serve as metal/metalloid-chelating agents under conditions of excess edaphic metal ions to alleviate metal stress. Anthocyanin accumulation might delay foliar senescence (accelerated in plants growing under macronutrient deficiency); this may be particularly advantageous to prolong plant survival and increase the possibility of reproductive success. Anthocyanins were demonstrated to play a role in the response of plants to environmental stress including drought, salt stress, light of high intensity, UV radiation, heavy metals, and low temperature [3,4]. Anthocyanins are present in nature mainly in the form of glycosides of aglycons (anthocyanidins) and carbohydrate moieties, sometimes containing additional substituents. More than 700 anthocyanins have been identified [1,5,6,7,8].

It has been suggested that anthocyanins consumed in food have many favorable health effects, often attributed to their antioxidant properties [9,10,11]. They have been reported to exert beneficial health effects in cardiovascular and neurodegenerative diseases, diabetes, and obesity, to improve fat metabolism and visual acuity, and to have anti-cancer and anti-inflammatory activity [7,8,9,10,12,13,14]. Nevertheless, the bioavailability of anthocyanins is low (1–2%) [9,15,16,17], making it highly unlikely for them to promote health as antioxidants. A more feasible reason for their beneficial effect may lie in the interaction of anthocyanins with critical proteins and signaling pathways. The antidiabetic action of anthocyanins was found to involve the facilitation of GLUT4 glucose transporter translocation, the suppression of dipeptidyl peptidase IV, the promotion of the secretion of glucagon-like peptide-1, the inhibition of protein tyrosine phosphatase 1B overexpression, and interaction with sodium-glucose co-transporter to delay glucose absorption [18], as well as an effect on the Wnt/β-catenin-WISP1 signaling pathways [19]. Anthocyanins’ prevention of liver fibrogenesis was found to be dependent on the inhibition of the expression of genes of the TGFβ/Smad/extracellular regulated protein kinase (ERK) signaling pathway [20]. The anti-inflammatory effect of anthocyanins was reported to be mediated by the suppression of the NF-κB/MAPKs signaling pathways [21]. The anti-adipogenic activity of anthocyanins was partially mediated by the AMPK pathway [22]. Inhibition of the phosphorylation of receptor tyrosine kinases (EGFR, VEGFR, Met receptor, and PDGFR), the down-regulation of downstream signaling cascades, and the inhibition of the activities of cAMP-specific phosphodiesterases, chymotrypsin-like proteasome, ornithine decarboxylase, cyclin-dependent kinases/cyclin complex, and the IκB kinase complex’s phosphorylation were reported to underlie the inhibition of cancer progression and the cancer-preventive action of anthocyanins [23]. The effect of anthocyanins on the intestinal microbiome is also important [22,24,25]. No negative effects of anthocyanin derivatives have been reported, even after ingestion of high doses [14,26]. Therefore, anthocyanins are used as safe food colorants and can be found not only in food derived from anthocyanin-containing raw materials but also in food colored with these compounds [27,28].

The characteristic feature of anthocyanins and anthocyanidins is their pH-dependent change in color. At an acidic pH, anthocyanins have a red color, which shifts to blue when pH increases to a neutral range. At a basic pH, anthocyanins are unstable and are oxidized into dark brown compounds [29,30]. Anthocyanins show considerable sensitivity to elevated temperatures [29,31,32] and oxidants [29,33].

The sensitivity of anthocyanins and anthocyanidins to conditions such as pH and temperature makes them promising indicators of food quality. Changes in the color of anthocyanin-containing foods caused by pH changes may be a measure of food deterioration [34]. “Intelligent” eco-friendly anthocyanin-containing foil and gels have been proposed to monitor the freshness of foods such as milk, meat, and fish [35,36,37].

Apart from pH changes, oxidation processes are also important among the causes of food deterioration. In our studies of the antioxidant properties of anthocyanidins and anthocyanins, we noted the new fluorescence band appearing upon oxidation of these compounds. We suggested that this new fluorescence band may be useful for the detection of oxidation in food products and biological samples [38]. This study aimed to provide a verification of this hypothesis by studying the fluorescence changes of endogenous anthocyanins during the storage of blueberry juice with air access and the cooking of blueberry homogenate and black carrot, along with those of delphinidin incubated with fried rapeseed oil.

## 2. Results

### 2.1. Oxidation of Blueberry Juice During Aerobic Storage

**Figure 1:**
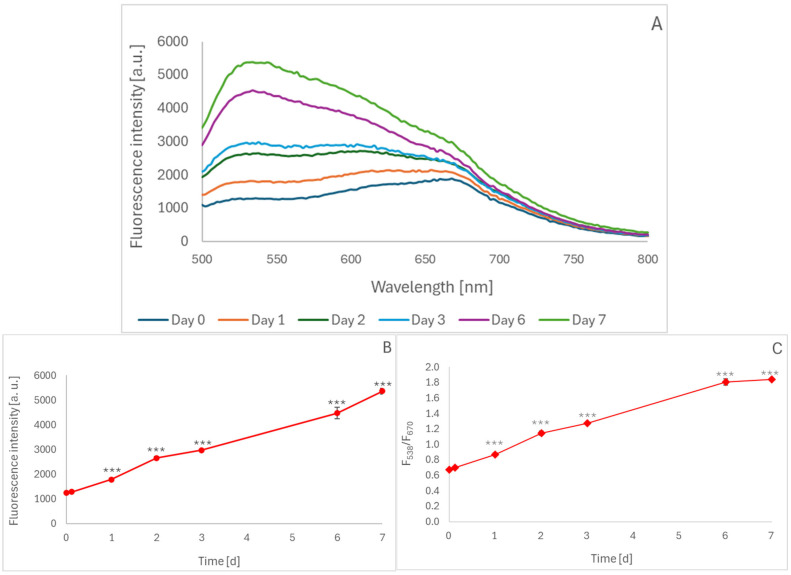
Emission spectra of blueberry juice incubated under air access for up to 7 days, excitation wavelength: 460 nm (**A**); fluorescence intensity of the juice at 538 nm as a function of incubation time (**B**); fluorescence intensity ratio (538 nm)/(670 nm) as a function of incubation time (**C**). In some cases, the standard deviation is lower than the data symbol size; *** *p* < 0.001 with respect to non-boiled samples.

Homemade blueberry juice, to which sodium azide was added to prevent bacterial growth, was incubated at room temperature under conditions allowing the access of air for up to 7 days, and it showed the appearance and gradual increase in fluorescence centered at about 538 nm when excited at 460 nm (Figure 1A). The intensity of this fluorescence peak increased progressively during 7-day incubation (Figure 1B). The ratio of fluorescence intensity at 538 nm to that at 670 nm (native fluorescence of cyanidins present in the juice), calculated from the spectra, also progressively increased over time (Figure 1C). This parameter seems more suitable for the analysis of unknown materials containing anthocyanins, as it is independent of anthocyanin concentration, in contrast to the intensity of the “oxidation band”.

### 2.2. Oxidation of Blueberry Homogenate During Cooking

**Figure 2:**
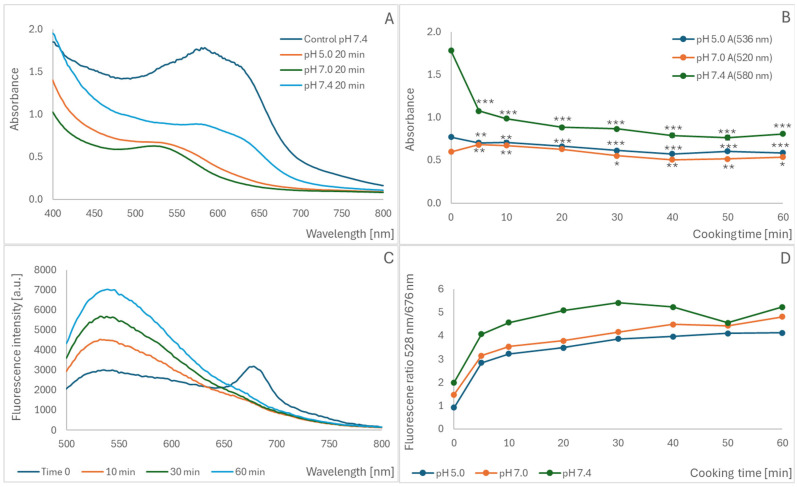
Effect of cooking blueberry homogenate for 20 min on the absorption spectrum of blueberry anthocyanins at various pH values (**A**); maximal absorbance as a function of the cooking time (**B**); effect of cooking blueberry homogenate at pH 5.0 for various times on the fluorescence spectrum of blueberry anthocyanins, excitation at 460 nm (**C**); effect of cooking time on the fluorescence intensity ratio at 528 nm/676 nm (**D**). Standard deviation values lower than the symbols in (**B**,**D**). * *p* < 0.05; ** *p* < 0.01; *** *p* < 0.001 (with respect to non-cooked samples); (**D**) *p* < 0.001 with respect to non-cooked samples in all cases.

The cooking of a blueberry homogenate resulted in a time-dependent decrease in absorbance at pH 7.4, 7.0, and 5.0 (Figure 2A,B). At pH 7.0, an increase was observed during the initial phase of cooking, followed by a decrease after 40–60 min (Figure 2B). A time-dependent increase in the fluorescence band centered at 520–540 nm for the excitation wavelength of 460 nm was observed during cooking (Figure 2C). The ratio of fluorescence intensities at 528 nm and 676 nm increased at all pH values studied, although the initial value of this ratio rose with increasing pH (Figure 2D). For all parameters studied, the most extensive changes took place during the initial cooking period (5–10 min) (Figure 2B,D), although the intensity of the fluorescence band centered at 530–540 nm also increased with longer cooking times (Figure 2C), reflecting progressive anthocyanin oxidation.

### 2.3. Oxidation of Black Carrot Anthocyanins During Cooking

**Figure 3:**
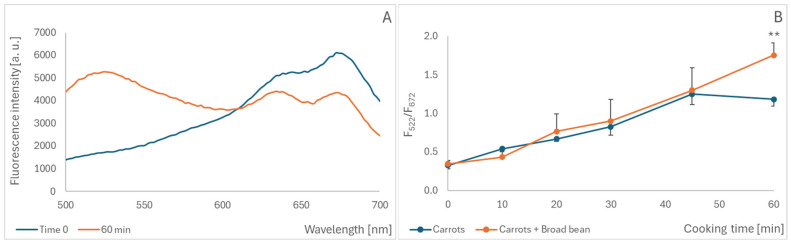
Effect of cooking black carrots on the fluorescence of endogenous anthocyanins. Fluorescence spectra of slices of black carrots, native and cooked for 60 min, excitation wavelength: 460 nm (**A**); the fluorescence intensity ratio at 532/672 nm of black carrots cooked for various times in the absence and presence of broad beans (**B**); ** *p* < 0.01.

When studying the effect of cooking on the oxidation of anthocyanins in black carrots, we took advantage of the possibility of performing a direct measurement of the fluorescence of whole slices in a plate reader, without the need to prepare extracts. Cooking slices of anthocyanin-rich black carrot also led to the appearance of a fluorescence band centered at 520–530 nm and a concomitant decrease in fluorescence in the range of 620–700 nm for the excitation wavelength of 460 nm (Figure 3A). The fluorescence intensity ratio 622 nm/672 nm increased progressively during heating, up to 50 min. The cooking of black carrot slices mixed with broad beans, a source of additional oxidants, increased the oxidation of anthocyanins after cooking for 60 min (for shorter cooking times, the difference was not statistically significant) (Figure 3B).

### 2.4. Oil Oxidation During Simulated Frying

**Figure 4:**
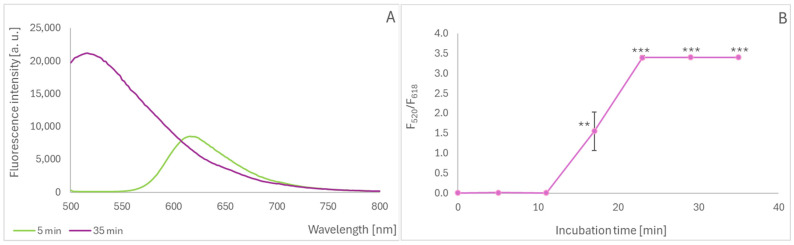

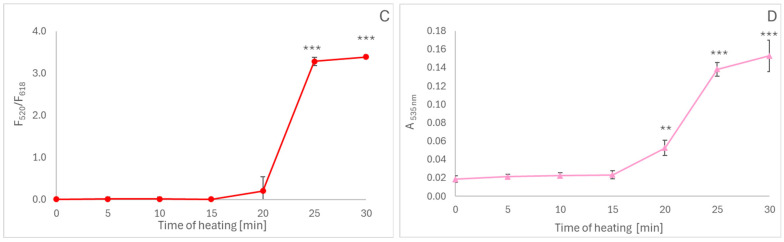
Oxidation of delphinidin incubated with heated rapeseed oil. Emission spectra of delphinidin incubated for 5 and 35 min with oil heated for 30 min; excitation wavelength of 360 nm (**A**); fluorescence intensity ratio at 520/618 nm as a function of the duration of incubation with oil heated for 30 min (**B**); fluorescence intensity ratio at 520/618 nm as a function of the duration of oil heating (**C**); Absorbance of thiobarbituric-reactive products in the oil as a function of the duration of heating (**D**). ** *p* < 0.01, *** *p* < 0.001.

Oils represent food products that do not contain anthocyanins but can be probed with hydrophobic anthocyanidins. When delphinidin was allowed to react with oil incubated at an elevated temperature to simulate frying, a gradual increase in the intensity of the new fluorescence band was observed (Figure 4A); this spectral change could also be characterized by a fluorescence intensity ratio at 520/618 nm. This ratio demonstrated that the reaction between the oxidants formed in the heated oil and delphinidin took about 20 min to complete (Figure 4B). Plotting the intensity ratio of delphinidin incubated with oil for 23 min as a function of the duration of heating showed that heating for 20–25 min caused massive oil oxidation (Figure 4C). The dependence of delphinidin oxidation on the duration of oil heating coincided with the formation of thiobarbituric acid-reactive products of lipid peroxidation (Figure 4D).

## 3. Discussion

Anthocyanins are fluorescent compounds, although their fluorescence quantum yield is low (4.1 × 10^−3^ for malvidin 3,5-diglucoside) [39]), mainly due to the efficient excited-state proton transfer to water [40]. Mainly for this reason, the fluorescence of anthocyanidins has not been extensively studied. Examples of such studies include the quenching of other fluorescent dyes by anthocyanins in the vacuoles [41], the intracellular localization of anthocyanidins by fluorescence lifetime microscopy [42,43], and the estimation of monomeric and polymeric anthocyanins in wine [44].

Nevertheless, to the best of our knowledge, no fluorescence studies of degradation products of anthocyanins have been performed, except for our previous paper [38]. We found that the appearance of the new fluorescence band, dependent on changes in the anthocyanidin aglycone moiety, is a general property of these compounds, as we observed it for six most common anthocyanidins and anthocyanins containing them (unpublished). Although the nature of the anthocyanin/anthocyanidin degradation product(s) is still being elucidated, this new fluorescence band is a reliable index of oxidation of these compounds by various oxidants. This fluorescence band did not appear in solutions of other flavonoids upon oxidation [38] or in slices of orange carrots, which are poor in anthocyanins, upon cooking.

Anthocyanin oxidation can be studied directly in food products containing sufficient amounts of endogenous anthocyanins, such as blueberries or black carrots. Malvidin is the main anthocyanidin found in blueberry anthocyanins, with malvidin-3-*O*-galactoside being the main anthocyanin [45]. However, a total of 54 anthocyanins were identified in blueberries, including cyanidin-3-*O*-glucoside, cyanidin-3-*O*-galactoside, cyanidin-3-*O*-arabinoside, delphinidin-3-*O*-glucoside, delphinidin-3-*O*-galactoside, petunidin-3-*O*-glucoside, petunidin-3-*O*-galactoside, petunidin-3-*O*-arabinoside, malvidin-3-*O*-glucoside, malvidin-3-*O*-galactoside, malvidin-3-*O*-arabinoside, peonidin-3-*O*-glucoside and peonidin-3-*O*-arabinoside [46,47], delphinidin-3-*O*-xyloside, delphinidin acetyl-galactoside, delphinidin acetyl-glucoside, cyanidin-3-*O*-xyloside, cyanidin acetyl-galactoside, cyanidin acetyl-glucoside, petunidin-3-*O*-xyloside, petunidin acetyl-galactoside, petunidin acetyl-glucoside, peonidin-3-*O*-xyloside, peonidin acetyl-galactoside, peonidin acetyl-glucoside, malvidin-3-*O*-xyloside, malvidin acetyl-galactoside, and malvidin acetyl-glucoside [48]. The main anthocyanins detected in black carrots (*Daucus carota* L. ssp. *sativus* var. *atrorubens* Alef.) were found to correspond to five cyanidin-based anthocyanins: cyanidin 3-*O*-xylosylglucosylgalactoside, cyanidin 3-*O*-xylosylgalactoside, and the sinapic, ferulic, and *p*-coumaric acid derivatives of cyanidin 3-*O*-xylosylglucosylgalactoside [49,50]. Another study also reported the presence of cyanidin 3-*O*-xylosylglucosylgalactoside, cyanidin 3-*O*-xylosylgalactoside, caffeic acid derivatives, and *p*-hydroxybenzoic acid derivatives of cyanidin 3-*O*-xylosylglucosylgalactoside [51].

We [38] and others [33,52] found previously that anthocyanidins and anthocyanins are oxidized by hydrogen peroxide. Hydrogen peroxide is generated during the cooking of vegetables [53,54], including black carrots, which generate more hydrogen peroxide than orange carrots [55], and thus, the oxidation of anthocyanins by the generated hydrogen peroxide should be expected during cooking. Such oxidation was indeed observed. Broad beans were found to generate high amounts of hydrogen peroxide [53], so the effect of cooking black carrots with broad beans was examined.

The obtained results demonstrate that the new fluorescence band, centered at 520–530 nm for the excitation wavelength of about 460 nm, appears and its intensity increases with the length of cooking of carrot slices. The presence of broad beans increased the oxidation of black carrot anthocyanins after 60 min of cooking. The formation of the new fluorescence band and a progressive increase in its intensity were also observed during the cooking of blueberry homogenate and the aerobic storage of blueberry juice.

Some food materials, like oils, do not contain anthocyanins. The oxidation of oils during storage or frying produces peroxides [56,57,58], and organic peroxides can also oxidize anthocyanidins and anthocyanins [38]. The incubation of delphinidin with thermally oxidized rapeseed oil induced oxidation of the anthocyanidin, as evidenced by the formation of an “oxidation band” of anthocyanidin fluorescence.

Although the appearance of the new fluorescence band can be easily monitored, its intensity is dependent on the concentration of anthocyanins/anthocyanidins in the examined material. A ratiometric parameter, independent of the anthocyanin concentration, is a more useful measure of the oxidation of these compounds. The data presented here demonstrate that the ratio of the fluorescence intensity at a wavelength corresponding to the fluorescence “oxidation band” to that typical of native anthocyanins/anthocyanidins may be such a parameter, which can be useful in the evaluation of oxidation processes in food products.

The possibility of measuring concentrated anthocyanin-rich solutions like juices or drinks and slices of solid materials using front-face fluorimetry, especially in fluorescence plate readers, makes such measurements broadly available. Perhaps a portable front-face fluorimeter could be constructed to measure the fluorescence of anthocyanin oxidation products, which would be even simpler but technically demanding. Taking into account the preferential accumulation of anthocyanins in the skin of fruits, such an approach would seem promising.

## 4. Materials and Methods

### 4.1. Material and Equipment

Sodium azide (CAS no. 26628-22-8; cat. no. 822335) and Tris base (CAS no. 7365-44-8; cat. no. 110695) were purchased from Merck (Poznań, Poland). Hydrochloric acid (CAS no. 7647-01-0; cat. no. 115752837) and trichloroacetic acid (CAS no. 115752837; cat. no. 115779700) were purchased from Chempur (Piekary Śląskie, Poland). Ethyl alcohol (CAS no. 64-17-5; cat. no. 396420113) was obtained from POCh (Gliwice, Poland). Thiobarbituric acid (CAS no. 504-17-6; cat. no. 36108.02) was provided by Serva (Heidelberg, Germany). Delphinidin chloride (CAS no. 528-53-0; cat. no. 0904 S) was obtained from EXTRA SYNTHESE (Genay, France). All other reagents, if not stated otherwise, were purchased from Merck (Poznan, Poland) and were of analytical grade. Distilled water was purified using a Milli-Q system (Millipore, Bedford, MA, USA). Black-flat-bottom 96-well plates (cat. no. 655209) or transparent flat-bottom 96-well plates (cat. no. 655101) (Greiner, Kremsmünster, Austria) were used for the assays. Fluorometric measurements were performed in a Spark multimode microplate reader (Tecan Group Ltd., Männedorf, Switzerland). Blueberries, black carrots, and broad beans were purchased in a local supermarket.

### 4.2. Oxidation of Anthocyanin-Rich Juice During Aerobic Incubation

Homemade blueberry juice was prepared: 25 g of fresh blueberries purchased in a local supermarket were added to 25 mL of water and 5 g of sucrose. The mixture was boiled for 10 min and filtered through a filter paper to obtain a clear juice, which was added to ca. 50 mg of sodium azide and incubated at room temperature (21 ± 1 °C) in a loosely covered flask to allow air access for up to 1 week. At various times, 200-µL aliquots of the juice were pipetted into wells of a black flat-bottom 96-well plate and fluorescence emission spectra were recorded using an excitation wavelength of 469 nm.

### 4.3. Cooking of Blueberry Homogenate

Blueberry (4 g) was added with 16 mL of 0.1 M acetate/citrate buffer, pH 5.0, 0.1 M sodium phosphate buffer, pH 7.0, or sodium phosphate buffer, pH 7.4, thoroughly homogenized, and boiled at 100 °C. Then the homogenate was centrifuged (5 min, 10,000× *g*) and the supernatant was used for analysis.

### 4.4. Cooking of Black Carrots

Disks of a diameter of about 5 mm, fitting wells of a 96-well plate, were cut from black carrots. Such disks were placed in Eppendorf tubes (2 per tube), mixed or not with about one quarter of a crushed broad bean, filled with distilled water to a volume of 2 mL, and the tubes were heated at 100 °C for up to 60 min. Then the carrot disks were withdrawn in 10-min intervals, dried with filter paper, placed in wells of a black plate, and their fluorescence was measured in a multiplate reader.

### 4.5. Oil Oxidation

Rapeseed oil “Kujawski” bought in a local supermarket was heated at a temperature of 246 °C, and samples were withdrawn at various time points. After cooling, 200 µL aliquots were pipetted into wells of a microplate and mixed with 20 µL of 10 mM delphinidin solution in ethanol. After 10 min of incubation, fluorescence spectra were measured as described above.

In parallel, the aldehyde of the heated oil was estimated with thiobarbituric acid. Briefly, 50 μL of the oil was added to 450 μL of 10 mM Tris-HCl buffer, mixed, and added with 500 μL of 10% trichloroacetic acid and 500 μL of 0.67% thiobarbituric acid in 0.1 M NaOH. After thorough mixing, the samples were incubated at 100 °C for 20 min. After cooling, absorbance was measured at 535 nm against a reagent blank.

## 5. Conclusions

Measurements of the ratio of fluorescence intensities at a band typical for anthocyanin/anthocyanidin oxidation (520–540 nm) to that typical for native anthocyanidins in the range of 600–700 nm, at the excitation wavelength of about 460 nm, may be a useful index of oxidation of food products rich in anthocyanins or enriched in anthocyanins/anthocyanidins.

## Data Availability

Dataset available on request from the authors.
